# Prognostic value of systemic inflammatory response syndrome and serum concentrations of acute phase proteins, cholesterol, and total thyroxine in cats with panleukopenia

**DOI:** 10.1111/jvim.15704

**Published:** 2020-01-24

**Authors:** Matteo Petini, Michele Drigo, Andrea Zoia

**Affiliations:** ^1^ Division of Internal Medicine San Marco Veterinary Clinic Padua Italy; ^2^ Department of Animal Medicine, Production and Health University of Padova Legnaro Italy

**Keywords:** diarrhea and vomiting, feline infection, infectious diseases, parvovirus

## Abstract

**Background:**

Feline parvovirus (FPV) is a common and potentially lethal infectious agent in cats.

**Objective:**

To assess the prognostic value of age, neuter status, serum concentrations of serum amyloid A (SAA), haptoglobin, cholesterol and total thyroxine (tT4), and the presence of systemic inflammatory response syndrome (SIRS) in cats with panleukopenia.

**Animals:**

Client‐owned cats with FPV infection diagnosed by a positive fecal ELISA test, positive PCR on feces or blood or both.

**Methods:**

Retrospective cohort study. The electronic medical database was searched for cats with FPV infection presented between January 2010 and January 2018. Cats were divided into survivors and nonsurvivors according to their survival status 28 days after hospital admission. The prognostic importance of each variable was investigated univariately and by multivariable Cox's proportional‐hazards regression. Finally, receiver operator characteristic (ROC) curve analysis was used to identify the best cutoff value for discriminating survivors from nonsurvivors for the statistically significant prognostic predictors identified by multivariable analysis.

**Results:**

Seventy cats were enrolled in the study. Multivariable analysis determined that only serum tT4 concentration at hospital admission was significantly (*P* = .01) associated with survival. A cutoff value of 0.82 μg/dL was identified by ROC curve analysis for serum tT4 concentration in discriminating survivors from nonsurvivors. Sensitivity at this cutoff was 73.9% and specificity was 82.9% (area under the curve, 0.783; 95% confidence interval, 0.668‐0.873; *P* < .0001).

**Conclusion and Clinical Relevance:**

Serum tT4 concentration at hospital admission has prognostic value in cats with FPV infection.

**Abbreviations**
APP: acute phase protein
CPV: canine parvovirus
CRP: C‐reactive protein
FPV: feline parvovirus
MLV: modified live vaccine
NTI: non‐thyroidal illness
SAA: serum amyloid A
SIRS: systemic inflammatory response syndrome
TSH: thyroid‐stimulating hormone
tT4: total tyroxine

## 1 INTRODUCTION

Parvovirus is an infectious agent in cats and in other mammalian species.^1^, ^2^ The virus is transmitted to susceptible animals by indirect contact with contaminated material.^1^ After initial replication in lymphoid tissue of the oropharynx, a viremia phase disseminates the virus to all body tissues.^1^, ^2^ Replication in the bone marrow and other lymphoid tissues commonly results in immunosuppression,^3^, ^4^ as a result of decreased leukocyte numbers and impaired T‐cell responsiveness.^4^ Often, patients die because of complications, such as secondary bacteremia, dehydration, and disseminated intravascular coagulopathy.^3^ Severe illness is more common in young and unvaccinated kittens in which the mortality rate can approach 90%.^2^, ^3^, ^5^

Compared with dogs,^7^, ^8^, ^9^, ^10^, ^11^, ^12^, ^13^, ^14^ only 2 studies evaluated the prognostic factors associated to feline parvovirus (FPV) infection in cats.^6^, ^15^ The clinical variables correlated with a higher risk of nonsurvival in FPV‐infected cats are the presence of lethargy, rectal temperature <37.9°C and low body weight at hospital admission.^15^ Moreover, in dogs with parvovirus infection, mortality is higher when criteria of systemic inflammatory response syndrome (SIRS) are met at admission,^9^ although standard criteria for SIRS in dogs are not yet universally accepted.^16^, ^17^, ^18^ The only clinicopathological variables associated with poor outcome in cats are leukopenia, lack of increase in leukocyte count after 3 days of hospitalization, thrombocytopenia, hypoalbuminemia, and hypokalemia.^6^, ^15^ In dogs affected by parvoviral infection, serum total thyroxine (tT4) and cholesterol concentrations are significantly lower at hospital admission in nonsurvivors compared with surviving dogs.^7^, ^8^, ^19^ Furthermore, 2 studies evaluating the prognostic value of acute phase proteins (APPs) in dogs with parvoviral enteritis indicated that C‐reactive protein (CRP) was higher in nonsurviving dogs^13^, ^14^ and positively associated with increased risk of death.^14^ However, the ability of CRP in predicting outcome was only moderately accurate.^14^

To our knowledge, no studies have investigated the role of serum tT4 concentration, SIRS, or the concentrations of any serum APPs in predicting the outcome of panleukopenia in cats. Moreover, no studies have established an association between serum cholesterol concentration at hospital admission and outcome.

Therefore, to improve knowledge about the prognosis of panleukopenia in cats, our aim was to assess the prognostic value of the presence of SIRS and the concentrations of serum amyloid A (SAA), haptoglobin, cholesterol, and tT4 in FPV‐infected cats at hospital admission.

## 2 MATERIALS AND METHODS

### 2.1 Study population and prognostic factors

A retrospective cohort study was planned enrolling cases by searching the electronic medical database P.O.A System‐Plus 9.0 for cats with panleukopenia presented to the “San Marco Veterinary Clinic” between January 2010 and January 2018. Cats were eligible for the study provided they had historical and clinical findings compatible with FPV infection and a definitive diagnosis of panleukopenia at hospital admission confirmed by direct ELISA assay (SNAP parvo test, Idexx Laboratories, Milan, Italy) on feces, positive PCR (MWG Biotech AG, Ebersberg, Germany) on feces or blood or both. Inclusion criteria were availability of a complete medical record, including signalment, history, and physical examination findings, plasma and serum samples obtained at the time of hospital admission, and a follow‐up evaluation at 28 days. The only exclusion criterion was vaccination against FPV within 4 weeks before hospital admission. Cats were divided into survivors and nonsurvivors according to their survival status 28 days after hospital admission. Cats euthanized because of worsening of their clinical condition or dead before day 28 after hospital admission as a result of FPV infection‐related complications were considered nonsurvivors.

Information on cats entering the study included sex (classified as intact or neutered), age, presence of SIRS at the time of admission, serum concentrations of SAA, haptoglobin, cholesterol and tT4, and survival status 28 days after hospital admission. Diagnosis of SIRS was made if the patient fulfilled at least 3 of the 4 SIRS criteria. The SIRS cutoff values were adopted from a previous study of cats: rectal temperature ≥39.7C° or <37.8C°, heart rate ≥225 beats/min or ≤140 beats/min, respiratory rate ≥40 breaths/min, and WBC count ≥19 500/μL or ≤5000/μL or band neutrophil fraction ≥5%.^20^

### 2.2 Analytical methods

All clinicopathological tests were performed at the “Laboratorio d'Analisi San Marco.” At hospital admission for all cats included in the study, a sample of 5 mL of blood was collected by jugular venipuncture for routine laboratory analysis. Two milliliters of blood were immediately transferred to a K_3_‐EDTA‐containing plastic tube for CBC performed by hematology analyzer (ADVIA 120 and 2120 hematology system, Siemens Healthcare GmbH, Erlangen, Germany), and 3 mL of blood were placed into a plain glass tube for routine serum biochemistry panel performed using an automated analyzer (Olympus AU 2700, Olympus Diagnostics, Hamburg, Germany), and including measurement of SAA, haptoglobin, and cholesterol concentrations. Serum tT4 concentrations were measured from the remaining serum, if available, on the same day of sample collection. Serum tT4 concentration was measured using an automated chemiluminescent immunoassay analyzer (Immunolite 2000; Siemens Healthcare GmbH). Clinicians managing each case were blinded to tT4 results and no kittens received thyroxine as part of their treatment.

All collection procedures were performed solely for the cat's benefit and for standard diagnostic and monitoring purposes. Informed written consent was obtained from all cat owners. Neither anesthesia nor euthanasia was required in any part of the study. All procedures complied with the European legislation on the protection of animals used for scientific purposes (Directive 2010/63/EU) and with the ethical requirement of Italian law (Decreto Legislativo 04/03/2014, n. 26). Accordingly, our study did not require authorization or ID protocol number.

### 2.3 Statistical analysis

Continuous data were assessed for normality of distribution using the Shapiro‐Wilk test. Normally distributed data were evaluated using mean ± standard deviation (SD), and non‐normally distributed data were reported as median and interquartile range (IQR, 25th and 75th percentiles). The prognostic importance of variables such as age, sex (classified as intact or neutered), presence of SIRS, serum concentrations of SAA, haptoglobin, cholesterol, and tT4 (as recorded at initial examination) was investigated by comparisons between survivors and nonsurvivors using the Mann‐Whitney test (“*U*” is the test statistic value), the Fisher exact test, the Yates‐corrected Chi‐square test (“χ^2^” is the test statistic value) and Student's test (“*t*” is the test statistic value), based on the nature and distribution of the variables. Cox's proportional‐hazards model was used to fit the association between survival status (at 28 days after hospital admission) as the dependent variable and the other prognostic variables with a *P* value <.05 when analyzed univariately as predictors. Finally, receiver operator characteristic (ROC) curve analysis was used to identify the best cutoff value for discriminating survivors from nonsurvivors for the prognostic variables that proved to be statistically significant in multivariable analysis.

For all statistical analyses, the significance level was set to α = .05.

## 3 RESULTS

### 3.1 Study population

One hundred twenty‐eight cats with a diagnosis of panleukopenia were presented to the “San Marco Veterinary Clinic” between January 2010 and January 2018. Of these, 70 cats were eligible for the study.

Of the 70 cats enrolled in the study, 47 (67%) were discharged alive (survivors) from the hospital with a median last follow‐up of 300 days (IQR, 60‐575 days). Among the survivors, 22 (47%) cats were female (7 sexually intact and 15 spayed) and 25 (53%) were male (15 sexually intact and 12 neutered); the median age of the survivor cats was 10 months (IQR, 7‐18 months) and their median duration of hospitalization was 5 days (IQR, 2‐7 days). Twenty‐three of 70 (33%) cats had a fatal outcome. All of these 23 cats died because of progression of the disease. None of the nonsurviving cats were euthanized because of worsening of their clinical condition or financial constraints. Among them, 11 cats (48%) were female (7 sexually intact and 4 spayed) and 12 (52%) were male (7 sexually intact and 5 neutered). The median age of these nonsurviving cats was 7 months (IQR, 3‐11 months). All nonsurviving cats died during hospitalization, except 1 that was discharged after 2 days of hospitalization at the owner's request and died at home 2 days later. The median survival time for nonsurviving cats was 3.5 days (IQR, 3‐7 days). Nonsurvivng cats were significantly (*U*, 314.5; *P* = .005) younger than surviving cats, whereas no significant difference (*P* = .36) in sex (classified as male intact, male neutered, female intact, or female neutered) was found between surviving and nonsurviving cats.

### 3.2 Prognostic factors

**Figure 1:**
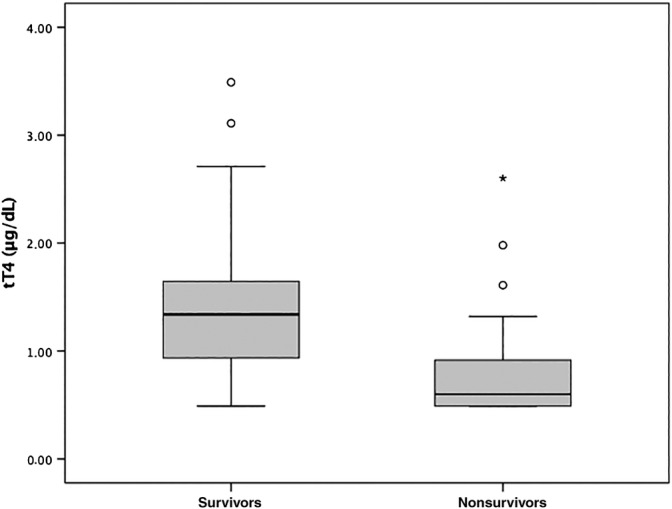
Box‐and‐Whisker plots for the concentration of serum total thyroxine at hospital admission in survivor (n = 47) and nonsurvivor (n = 23) cats with feline panleukopenia and a follow‐up time of 28 days. The bounds of the boxes are the 1st (the lower) and 3rd (the upper) quartiles; the black line across the boxes represents the median (2nd quartile). Whiskers correspond to data still within 1.5 interquartile range from the 1st and the 3rd quartiles. Circles and stars are outlier and extreme outlier values (more than 1.5 and 3 interquartile range away from the closest end of the box, respectively)

At hospital admission, a statistically significant difference (χ^2^ = 8.02; *P* = .005) was found in the number of cats fulfilling SIRS criteria between surviving (9/47, 19%) and nonsurviving (12/23, 52%) cats. The SAA concentration (reference interval, 0.1‐0.5 μg/dL) was significantly lower (*U* = 749; *P* = .009) in surviving (median, 83.3 μg/dL; IQR, 0.1‐143.4 μg/dL) compared with nonsurviving cats (median, 138.3 μg/dL; IQR, 96.0‐167.1 μg/dL). Serum haptoglobin concentrations (reference interval, 18‐74 mg/dL) were significantly lower (*t* = −3.24; *P* = .002) in surviving (mean, 136.13 ± 69.80 mg/dL) compared with nonsurviving cats (mean, 190.09 ± 55.25 mg/dL). Serum cholesterol concentrations (reference interval, 95‐210 mg/dL) were not significantly different (*t*, 0.838; *P* = .40) in surviving (mean, 142.77 ± 36.48 mg/dL) compared with nonsurviving cats (mean, 134.22 ± 46.77 mg/dL). Finally, serum tT4 concentrations (reference interval, 1.21‐3.29 μg/dL) were significantly higher (*t*, 3.546; *P* = .001) in surviving (mean, 1.38 ± 0.66 μg/dL) compared with nonsurviving cats (mean, 0.84 ± 0.56 μg/dL; Figure 1).

Results of multivariable Cox proportional hazards regression indicated that serum tT4 concentration at initial examination was the only factor significantly associated with survival (hazard ratio, 0.26; *P* = .01) after controlling for the other predictors (Table 1).

**Table 1:** Multivariable Cox proportional hazards regression investigating the association between the 28‐day mortality and the predictor variables with a *P* value <.05 in univariate analysis

Risk factor	Hazard ratio	95% CI	*P* value	Wald statistics
tT4	0.26	0.09‐0.76	.01	6.060
Age	0.94	0.88‐1.01	.11	2.555
SIRS	1.07	0.41‐2.81	.89	0.017
Haptoglobin	1.00	0.99‐1.01	.26	0.005
SAA	1.00	0.99‐1.01	.42	0.636

Abbreviations: SAA, serum amyloid A; SIRS, systemic inflammatory response syndrome; tT4, total thyroxine.

**Figure 2:**
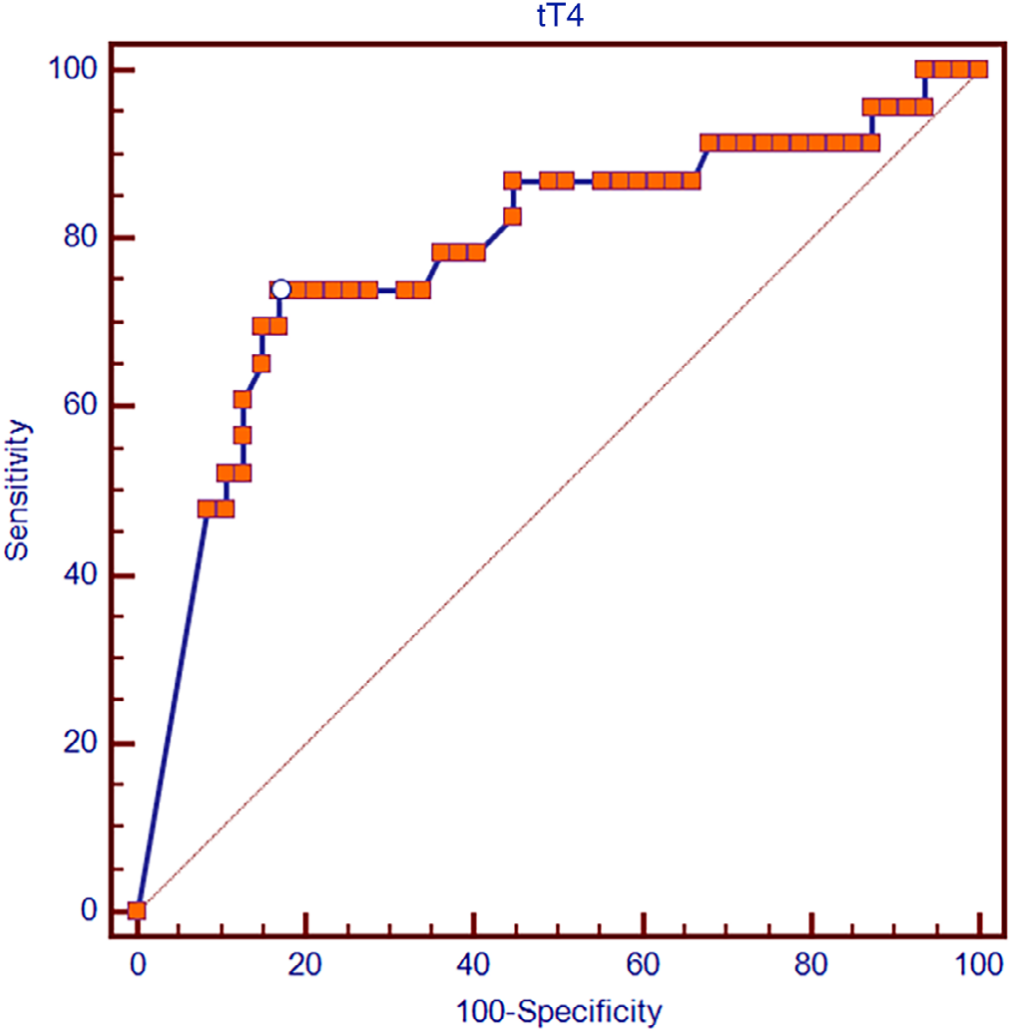
Receiver operating characteristic curve for the use of total tyroxine concentration at initial examination for discriminating 28 days survivor from nonsurvivor cats with feline panleukopenia. The white circle corresponds to the Youden index that identifies the value providing the greatest sum of sensitivity and specificity

The optimal cutoff value identified by ROC curve analysis for serum tT4 concentration (ie, the value that maximized the sum of sensitivity and specificity [Youden index] in discriminating survivors from nonsurvivors) was 0.82 μg/dL. Serum tT4 concentration ≤0.82 μg/dL had a sensitivity of 73.9% and a specificity of 82.9% (AUC, 0.783; 95% CI for the AUC, 0.668‐0.873; *P* < .0001) to predict mortality (Figure 2).

## 4 DISCUSSION

In our study, multivariable analysis showed that only serum tT4 concentrations at hospital admission could predict fatal outcome in cats with FPV infection. Presence of SIRS, serum cholesterol concentration, and concentrations of all APPs evaluated at hospital admission were not associated with death in these cats with panleukopenia.

Critical illness is characterized by changes in thyroid hormone concentrations even in the presence of normal thyroid function, and this state is defined as non‐thyroidal illness (NTI).^21^ This condition has been attributed to 3 general factors: alterations in deiodinase activity, alterations in thyroid‐stimulating hormone (TSH) secretion, and alterations in thyroid hormone binding to serum proteins. Recently, other mechanisms such as an altered transport of the thyroid hormones into the tissue and altered nuclear thyroid hormone receptor activity have been proposed.^22^ In human medicine, the existence of a correlation between low serum T4 concentrations and poor outcome is recognized in critically ill patients.^23^, ^24^, ^25^ There are some concerns about thyroid hormone replacement in human patients with NTI, but to date a consensus on this topic is lacking.^23^ Critical illness is a cause of NTI in both dogs and cats. In dogs, many studies have evaluated the prognostic implication of thyroid hormones in sick patients.^8^, ^19^, ^26^, ^27^, ^28^, ^29^ It has been determined that serum tT4 concentrations in cats with NTI progressively decrease with the worsening of the disease.^30^ Another study in cats with various diseases not associated with the thyroid gland showed that serum tT4 concentrations are inversely correlated with mortality.^31^ However, to our knowledge, no studies in cats have evaluated the prognostic value of thyroid hormone concentrations for a specific individual disease. Our results showed that serum tT4 concentrations at hospital admission were associated with the outcome of FPV‐infected cats. This observation is consistent with the findings of the previously mentioned studies.

In addition to the primary aim of our study, we also investigated the possible association of other predictors such as age and sex (classified as intact or neutered) with survival, although the 2 groups were not matched for these variables. In fact, kittens are considered to be more susceptible to FPV infection with highest reported morbidity and mortality between 3 and 5 months of age,^2^, ^4^ but that assumption is based on expert opinion and has not been demonstrated by observational studies. In our study, age was shown in univariate analysis to be a prognostic factor but in the multivariable analysis, it was no longer associated with outcome. Therefore, this result is in contrast with what is generally suggested,^2^, ^4^ but it is in accordance with another study.^15^ Conversely, sex in panleukopenia does not seem to have a prognostic role.^6^, ^15^ In fact, in the exploratory univariate analysis, sex (classified as intact or neutered) did not correlate with prognosis in our study, in accordance with previous reports in cats.^6^, ^15^

Criteria of SIRS have not been well established in cats, with different inclusion criteria reported.^20^, ^32^ For the purpose of our study, diagnosis of SIRS was made according to the criteria recently used in cats.^20^, ^33^ However, to increase the specificity of SIRS criteria, we chose the presence of ≥3 criteria, instead of ≥2, as suggested by a previous study of cats.^32^ Considering the disruptive effects of FPV on intestinal epithelial cells,^34^ SIRS can occur or be enhanced by gram‐negative endotoxemia, which is a common sequela of systemic FPV infection. This hypothesis is supported by the milder clinical signs exhibited by specific pathogen‐free kittens experimentally infected by FPV.^4^ When present, SIRS could contribute to the occurrence of NTI in this subset of patients. Therefore, despite not being a significant predictor in the multivariable prognostic model, SIRS still may play an indirect role in determining final outcome and contribute to the presence of NTI. It is possible that different SIRS criteria may produce different results. Considering the potential prognostic value of SIRS status, we believe that a consensus on the final definition of this condition in cats is needed.

Despite the wide use of APPs in the clinical feline practice, there is little evidence of their prognostic role in sick cats.^35^, ^36^ Recently, the potential diagnostic utility of SAA concentrations measured at the time of hospital admission was investigated for discrimination between infectious and noninfectious SIRS in critically ill cats.^35^ No association between SAA concentrations at hospital admission and final outcome was identified.^35^ Similarly, our study did not identify a prognostic role for serum concentrations of APPs at hospital admission in predicting the outcome in cats with FPV infection. It is possible that longitudinal monitoring, rather than a single APP determination, may better predict outcome as suggested by studies in dogs.^37^, ^38^

We did not find any difference in serum cholesterol concentrations between surviving and nonsurviving cats with panleukopenia. This result is in agreement with another study.^15^ However, in canine parvovirus (CPV)‐infected dogs, nonsurvivors have a significantly lower serum cholesterol concentrations than do surviving dogs.^7^ A possible explanation could derive from the different biological behavior of parvoviruses in dogs and cats. In our opinion, clinical evidence suggests that in cats gastrointestinal signs caused by FPV are less common and milder when present, as compared to CPV infection in dogs. In fact, parvoviral infection in dogs historically is known as canine parvoviral enteritis,^7^, ^10^, ^12^, ^13^, ^39^, ^40^, ^41^, ^42^, ^43^ whereas parvoviral disease in cats traditionally is named feline panleukopenia.^2^, ^6^, ^15^, ^44^ Thus, because hypocholesterolemia is also a marker of severity of gastrointestinal signs, it is not surprising that dogs with hypocholesterolemia, and possibly a more severe form of the disease, also have a worse outcome.^7^

Several limitations could have affected our study. First, we excluded both cats with inconsistent vaccination history and cats vaccinated within 4 weeks before hospital admission. We used this criterion because cats receiving modified live vaccines (MLV) can shed the vaccine virus and consequently false‐positive results of direct ELISA tests on fecal samples can occur up to 2 weeks after vaccination.^45^, ^46^ However, in dogs vaccinated against CPV with MLV, a secondary viremia or fecal shedding was demonstrated for >2 weeks, thus potentially interfering with the PCR test.^47^ Although a similar study in cats has not been performed, we excluded cats vaccinated within 4 weeks before the onset of clinical signs compatible with FPV infection to limit the inclusion of false‐positive cats. Second, because of the retrospective nature of the case selection, it was not possible to ensure a standard protocol for blood sampling, thus leading to the exclusion of some cases from the study. In fact, samples were not collected if the patient's health condition appeared too critical, in case of lack of patient compliance, or because of financial constraints of the owner. Third, during the 8 years of the study, some changes in treatment procedures could have occurred, affecting the development of a standard protocol for treatment of panleukopenia and therefore potentially influencing the final outcome of each cat. An additional limitation restricting the statistical power of the study could lie in the low number of nonsurviving cats compared with the survivors. Despite all the limitations of a retrospective study, we believe that the multivariable approach used and the lack of a unique and systematic cause of exclusion did not substantially affect the robustness of the results.

## 5 CONCLUSIONS

We showed that serum tT4 concentration at hospital admission in cats with FPV infection could be used as a prognostic factor in predicting disease outcome. Further studies with serial monitoring of APPs and SIRS status are needed to better evaluate their prognostic relevance in this common infectious disease of cats.

## CONFLICT OF INTEREST DECLARATION

Authors declare no conflict of interest.

## OFF‐LABEL ANTIMICROBIAL DECLARATION

Authors declare no off‐label use of antimicrobials.

## INSTITUTIONAL ANIMAL CARE AND USE COMMITTEE (IACUC) OR OTHER APPROVAL DECLARATION

Authors declare no IACUC or other approval was needed.

## HUMAN ETHICS APPROVAL DECLARATION

Authors declare human ethics approval was not needed for this study.
